# Pseudoaneurysm of thoracic aorta presenting as inappropriate sinus tachycardia: a case report

**DOI:** 10.1186/s13256-019-2167-8

**Published:** 2019-08-03

**Authors:** Zahra Azizi, Pouria Alipour, Maria Terricabras, Yaariv Khaykin

**Affiliations:** 1 Southlake Regional Health Centre, 602-581 Davis Drive, Newmarket, Ontario L3Y 2P6 Canada; 2 York University Faculty of Heath, Toronto, Ontario Canada

**Keywords:** Inappropriate sinus tachycardia, Pseudoaneurysm of thoracic aorta, Chronic cough

## Abstract

**Background:**

Pseudoaneurysm of thoracic aorta as a complication of blunt trauma to the chest, can present with a variety of symptoms due to mass compression effect. Here we report the first pseudoaneurysm of thoracic aorta presenting with chronic cough and inappropriate sinus tachycardia. The purpose of this case report is to highlight pseudoaneurysm of thoracic aorta as a rare differential diagnosis for inappropriate sinus tachycardia.

**Case presentation:**

Here we report a case of 29-year-old white woman, a nurse, with history of a motor vehicle accident. She initially presented to medical attention with inappropriate sinus tachycardia 2 years following the motor vehicle accident during her pregnancy. Six years later she underwent sinoatrial node modification after failing a number of medications. Days prior to the ablation she developed a mild cough which became constant within a week following ablation. A computed tomography scan of her chest performed as part of a workup revealed an outpouching of the inferomedial aspect of the aortic arch, which was compressing her left main bronchus. She underwent arch repair surgery and recovered without complications. Four years later she presented with significant symptomatic sinus bradycardia requiring pacemaker placement.

**Conclusions:**

This is the first reported case of thoracic pseudoaneurysm of aorta presenting with inappropriate sinus tachycardia due to compression of the vagal nerve and cough as a result of the left main bronchus compressive effect; it highlights the importance of considering structural abnormalities in a differential diagnosis of inappropriate sinus tachycardia before any interventions.

## Introduction

Pseudoaneurysm of thoracic aorta (PTA) can occur due to blunt trauma to the chest, cardiothoracic surgery, and connective tissue disorders [1, 2]. This condition is usually asymptomatic and is incidentally identified on imaging studies. Depending on size and location of aneurysms, the symptoms if present may vary from dysphagia, hemoptysis, dyspnea, hoarseness, to recurrent pneumonitis [2, 3]. There are few cases that report chronic cough due to compression of left main bronchus as a rare symptom of the aortic pseudoaneurysm [2–4]. Here we report the first case of PTA presenting with chronic cough and inappropriate sinus tachycardia (IST). The purpose of this case report is to highlight PTA as a rare differential diagnosis for IST.

## Case presentation

A 29-year-old white woman, a nurse, presented initially with sudden episodic palpitations in the absence of physical or emotional stress, which started during her pregnancy 6 years prior to visit and progressed to incessant rapid heart rates throughout the day. Her workup was negative for deep vein thrombosis (DVT), pulmonary embolism, thyroid dysfunction, and adrenal dysfunction. She had normal cardiac echocardiography. The results of a chest X-ray, ventilation–perfusion (V/Q) scan, as well as pulmonary function test (PFT) were normal. Her 24-hour Holter showed average heart rate of 118 beats per minute (bpm) with peak heart rate of 160 despite sotalol 80 mg twice a day. Her past medical history was positive for tobacco smoking, psoriatic arthritis, tonsillectomy, and a motor vehicle accident (MVA) 2 year prior to the initial onset of tachycardia.

**Fig. 1:**
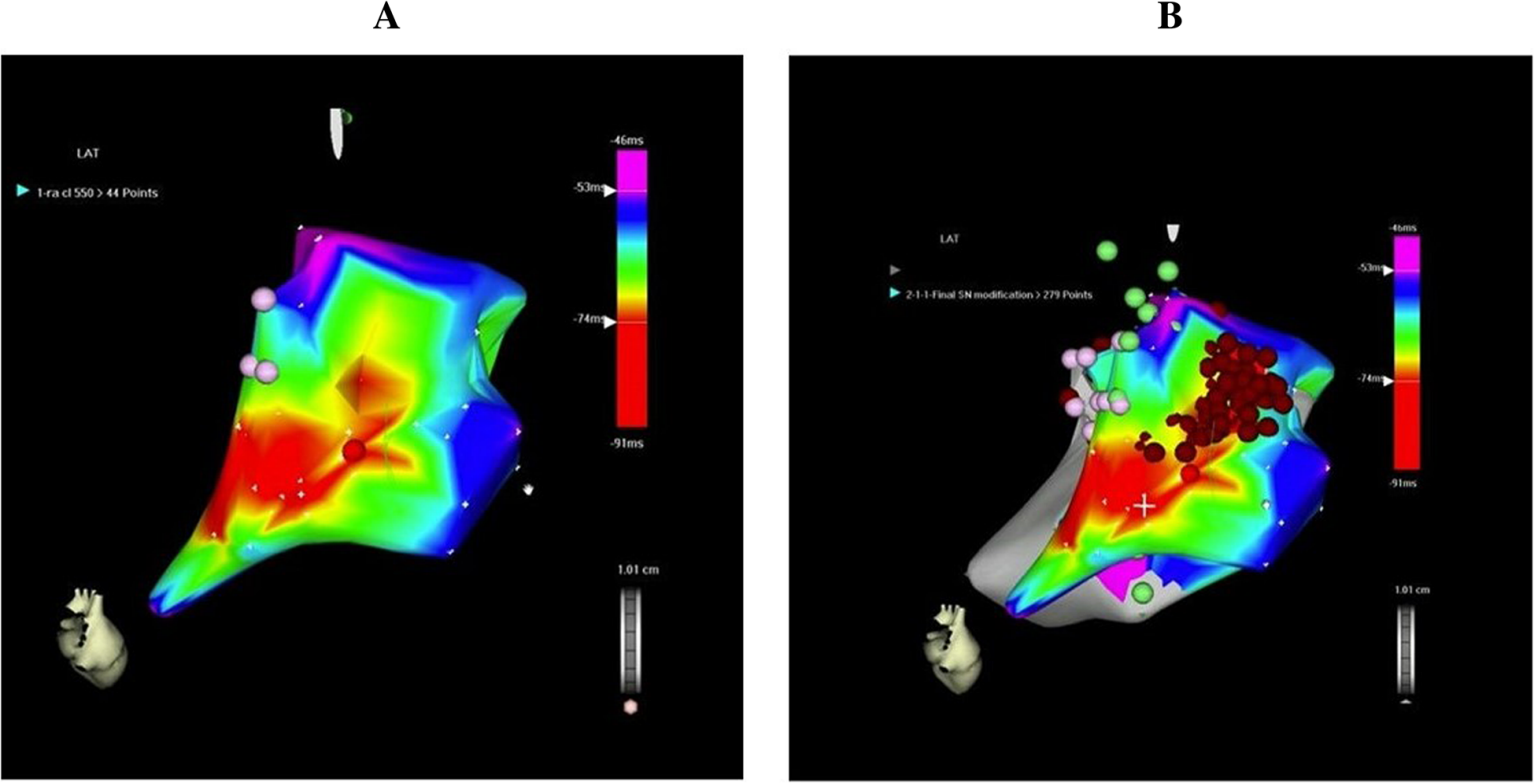
Sinoatrial node is a long structure with slower more caudal portion of the node producing a flat or inverted p-wave in the inferior leads and faster more cranial portion of the node producing more upright p-waves. **a** Baseline electroanatomic map of sinus node map pre-isoproterenol at a baseline rate around 110 beats per minute. **b** Map following ablation: note that ablation was delivered at a more cranial portion of the sinus node

**Fig. 2:**
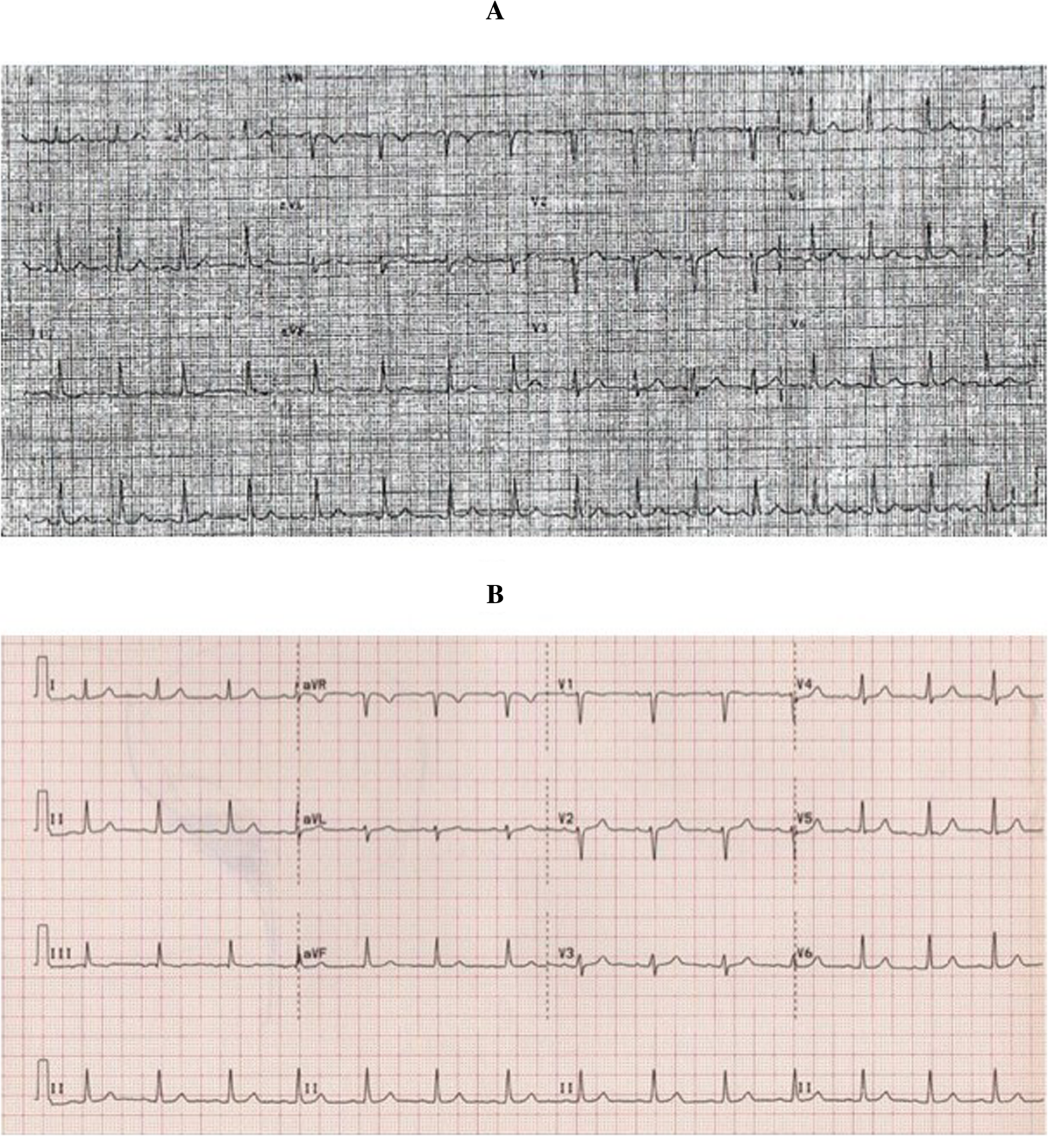
**a** Patient baseline electrocardiogram before ablation. **b** Patient’s electrocardiogram after ablation; notice flattening/inversion of the p-waves in the inferior leads

Since she had failed attempts at aggressive hydration, propranolol, atenolol, sotalol, and selective serotonin reuptake inhibitors (SSRIs), she was offered a sinoatrial (SA) node modification procedure using three-dimensional electroanatomic mapping. On the day of ablation, she presented with a mild cough. An electrophysiology study including programmed ventricular and atrial stimulation showed no evidence for dual atrioventricular (AV) nodal physiology and accessory pathway conduction and no evidence for any inducible ventricular or atrial arrhythmias. She had a heart rate of 110 bpm at baseline that went up to 160 bpm on 2 μg/minute of isoproterenol and 180 bpm on 4 μg/minute of isoproterenol. An electroanatomic map of her right atrium and the SA node was constructed at rest and on isoproterenol (Fig. 1a, b). The course of the phrenic nerve was mapped using high output pacing. After sinus node (SN) modification, our patient’s heart rate was 50–60 off isoproterenol with flat to inverted p-waves in the inferior leads (Fig. 2a, b). There was no visible injury to the phrenic nerve.

Following ablation, our patient developed symptoms of pericarditis, pleuritic pain radiating to her left shoulder, and worsening cough, particularly when lying down with some orthopnea. Her jugular venous pressure was normal. She was initially treated with diclofenac 50 mg twice a day, Tylenol (acetaminophen), and levofloxacin 500 mg daily. After 2 days, she presented with nausea, vomiting, loose stool, orthopnea, and worsening cough when lying down. A chest X-ray showed a small left pleural effusion and her electrocardiogram (ECG) was unchanged from the last ECG. Cardiac echocardiography remained normal with no evidence of pericardial effusion or other explanation for her symptoms. Doppler ultrasound (US) of her legs showed no DVT and her V/Q scan was negative for pulmonary embolism and chest fluoroscopy again confirmed normal phrenic nerve function. Her blood work was unremarkable.

**Fig. 3:**
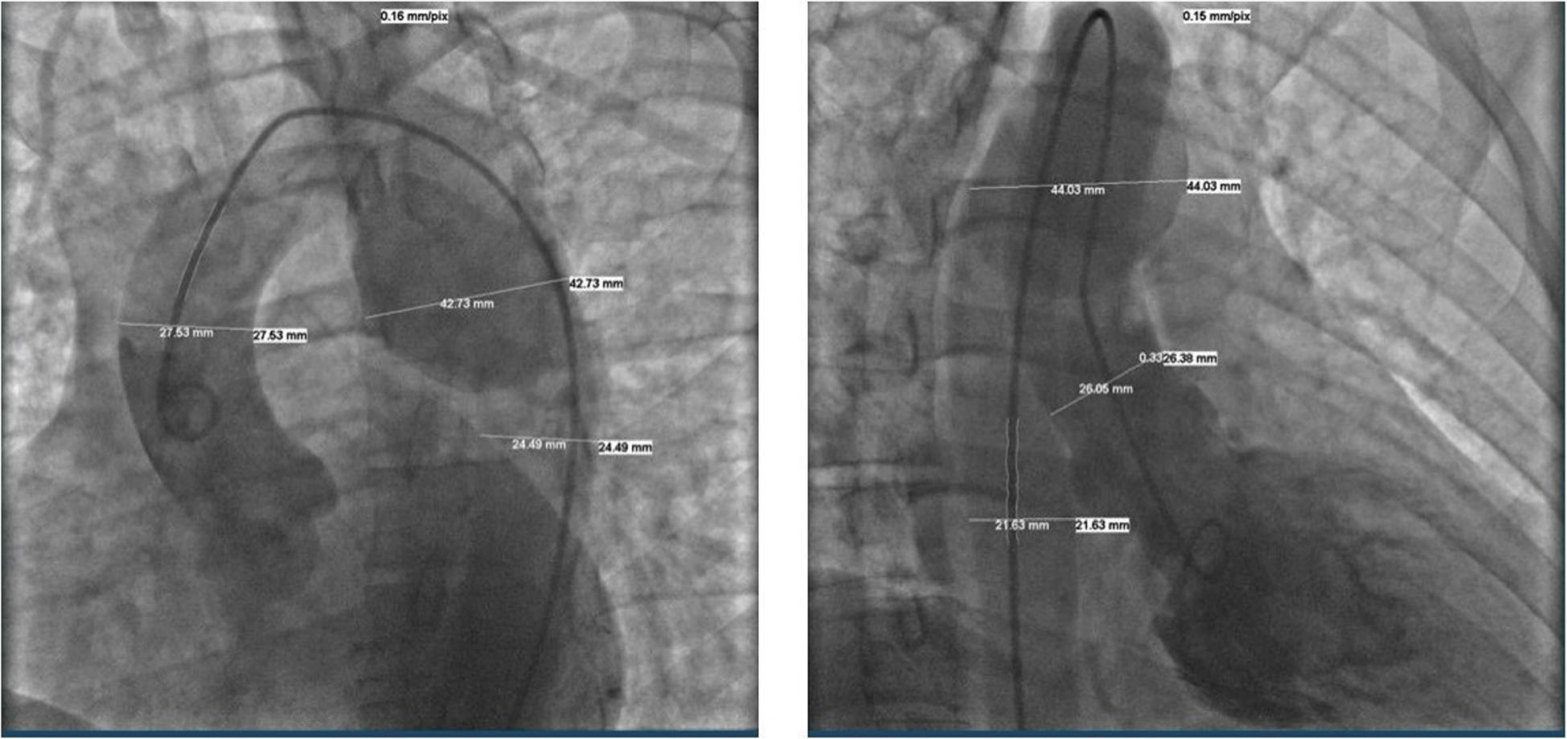
Angiography of aortic arch confirming pseudoaneurysm of aorta

**Fig. 4:**
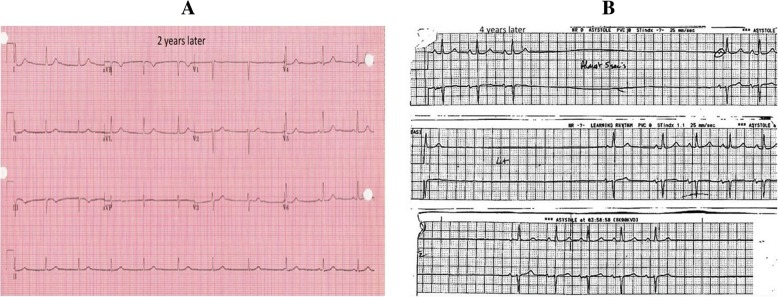
Patient’s electrocardiogram. **a** Two years after ablation. **b** Four years after ablation

A computed tomography (CT) scan of her chest showed an outpouching of the inferomedial aspect of the aortic arch 3.8 × 3.9 cm in size which was compressing her left main bronchus. This was confirmed as a pseudoaneurysm by angiography (Fig. 3). She underwent resection and graft repair of the descending thoracic aorta with no complications. All her symptoms resolved after surgery and she returned to work. Four years following ablation she presented to our emergency room (ER) feeling weak and dizzy. She was found to have recurrent episodes of sinus arrest, with pauses up to 3 seconds followed by a nodal escape beat (Fig. 4a, b). She was not being treated with any medications known to suppress SN function at this time. She was admitted with diagnosis of sick sinus syndrome and underwent permanent pacemaker implantation.

## Discussion

Autonomic innervation of heart includes vagal (parasympathetic) and sympathetic fibers. IST is characterized by SN-driven rhythm at a rate elevated over 100 bpm or rapidly accelerating to over 100 bpm without identifiable physiological or emotional stressors [5, 6]. Potential mechanisms may include: an ectopic atrial focus close to the SA node; increased sympathetic tone or decreased response to vagal stimulation due to reduced sensitivity of the muscarinic receptors [7]; depressed efferent vagal activation [8]; non-muscarinic, non-adrenergic, or vagally mediated mechanisms [9]; neurohormonal modulation [10]; hypothalamic paraventricular nucleus stimulation [11]; and intrinsic abnormality of the SA node [5, 6].

A variety of symptoms have been described to occur due to the local mass effect of a thoracic aortic aneurysm including cough as a result of tracheal or bronchial compression, and hoarseness due to stretching of the recurrent laryngeal nerve, dysphagia due to esophageal compression, and chest pain as an early sign of rupture or dissection [1–4, 12]. This case shows a combination of cough and IST as presenting complaints related to the aortic pseudoaneurysm, probably the result of an earlier MVA. Changes in hemodynamics and soft tissues during pregnancy may have led to further dilatation of the aneurysm with impingement on the vagal nerve fibers constituting the cardiac plexus just in front of the tracheal bifurcation and, therefore, right under the expanding aneurysm [13]. Further enlargement of the lesion after ablation may have been related to the isoproterenol infusion used to increase the heart rate to 150 bpm during mapping and ablation. This may have accelerated the process already under way, leading to tracheobronchial irritation by the enlarging aneurysm. Finally, 4 years after the aortic repair, and removal of the mass effect on the vagal nerve, our patient developed sick sinus syndrome which required a permanent pace maker possibly due to vagal re-innervation.

## Conclusion

This is the first reported case of thoracic pseudoaneurysm of the aorta presenting with IST due to compression of the vagal nerve and cough as a result of the left main bronchus compressive effect; it highlights the importance of considering structural abnormalities in a differential diagnosis of IST before any interventions.

### Key clinical message

This is the first reported case of PTA presenting with IST due to compression of the vagal nerve and cough as a result of left main bronchus compression; it emphasizes the importance of considering structural abnormalities in a differential diagnosis of IST before any interventions.

## Data Availability

Not applicable.
